# The Statistical Meaning of Kurtosis and Its New Application to Identification of Persons Based on Seismic Signals

**DOI:** 10.3390/s8085106

**Published:** 2008-08-27

**Authors:** Zhiqiang Liang, Jianming Wei, Junyu Zhao, Haitao Liu, Baoqing Li, Jie Shen, Chunlei Zheng

**Affiliations:** Shanghai Institute of Micro-system and Information Technology, Chinese Academy of Sciences, 200050, Shanghai, P.R. China

**Keywords:** Kurtosis, peakedness, tail, seismic signal, person identification

## Abstract

This paper presents a new algorithm making use of kurtosis, which is a statistical parameter, to distinguish the seismic signal generated by a person's footsteps from other signals. It is adaptive to any environment and needs no machine study or training. As persons or other targets moving on the ground generate continuous signals in the form of seismic waves, we can separate different targets based on the seismic waves they generate. The parameter of kurtosis is sensitive to impulsive signals, so it's much more sensitive to the signal generated by person footsteps than other signals generated by vehicles, winds, noise, etc. The parameter of kurtosis is usually employed in the financial analysis, but rarely used in other fields. In this paper, we make use of kurtosis to distinguish person from other targets based on its different sensitivity to different signals. Simulation and application results show that this algorithm is very effective in distinguishing person from other targets.

## 1. Introduction

Persons or other targets moving on the ground generate continuous impacts which propagate in the form of seismic waves that can be measured by geophones or seismic sensors. The signal generated by a person's footsteps can be distinguished from the signals generated by other targets, based on their impulsive nature.

Many previous papers have focused on feature extraction and classifier design. These methods are so complicated and lacking in robustness, that it is impractical to apply them to common applications. In [1] a new feature extraction algorithm based on the mel-cepstrum analysis was investigated, but it can only be used to some special environments. A novel target classification method by means of a microaccelerometer has been described [2]. It is also particular to some special environments and complicated. In order to make these methods applicable to new environments, it is necessary to train the classifier again and again. In [3] the characteristics of people's footsteps signature were examined, but no effective algorithm to identify persons from other targets was shown. Paper [4] proposes a new feature extraction method based on psycho-acoustics parameters to recognize people's footsteps, but it's impossible to apply the algorithm widely as acoustics signal is easily disturbed.

From above, we can see that there are more or less faults in the existing methods used in person recognition. In this paper, we provide an algorithm using the parameter of kurtosis which shows more simpleness and robustness. The remainder of this paper is organized as follows: section 2 describes the statistical meaning of kurtosis. Section 3 lists and discusses the simulation results of the algorithm using kurtosis which is applied to recognize person footsteps. Section 4 gives the conclusion and predicts future work.

## 2. The statistical meaning of kurtosis

Karl Pearson [5] defined a distribution's degree of kurtosis as:

$$\eta =\beta_{2}-3$$

where

$$\beta_{2}=\frac{\sum {\left(X-\mu \right)}^{4}}{n\sigma^{4}}$$

X denotes the sequence of inputs, *μ* represents the mean value of X, *σ* is referred to the variance of X and n the length of input sequence X. The expected value of the distribution of $Z=\frac{X-\mu }{\sigma }$ scores which have been raised to the fourth power. *β*_2_ is often referred to as “Pearson's kurtosis”, and *β*_2_ − 3 (often symbolized with *γ*_2_, that is *γ*_2_ =*β*_2_ −3) as “kurtosis excess” or “Fisher's kurtosis”.

An unbiased estimator [6-8] for *γ*_2_ is

$$g_{2}=\frac{n(n+1)\sum Z^{4}}{\left(n-1)(n-2)(n-3\right)}-\frac{{3(n-1)}^{2}}{\left(n-2)(n-3\right)}.$$

For large sample sizes (n>1000), *g_2_* may be distributed approximately normally, with a standard error of approximately $\sqrt{24/n}$.

Pearson [5] introduced kurtosis as a measure of how flat the top of a symmetric distribution is when compared to a normal distribution of the same variance. He referred to more flat-topped distributions (*γ_2_<* 0) as “platykurtic”, less flat-topped distributions (*γ_2_>* 0) as “leptokurtic”, and equally flat- topped distributions as “mesokurtic” *(γ_2_* ≈0). Kurtosis is actually more influenced by scores in the tails of the distribution than scores in the center of a distribution [9]. Accordingly, it is often appropriate to describe a leptokurtic distribution as “fat in the tails” and a platykurtic distribution as “thin in the tails”. Platykurtic curves have shorter ‘tails’ than the normal curve of error and leptokurtic longer ‘tails’.

Moors [10] demonstrated that *β*_2_ = *V*(*Z*^2^) + 1. Accordingly, it may be best to treat kurtosis as the extent to which scores are dispersed away from the shoulders of a distribution, where the shoulders are the points where *Z^2^*= 1, that is, *Z* = ±1. Balanda and MacGillivray [11] wrote “it is best to define kurtosis vaguely as the location- and scale-free movement of probability mass from the shoulders of a distribution into its centre and tails”. If one starts with a normal distribution and moves scores from the shoulders into the center and the tails, keeping variance constant, kurtosis will increase. The distribution will likely appear more peaked in the center and fatter in the tails, like a Laplace distribution (*γ*_2_= 3).

Let us denote p(x) the probability density function (pdf) of a random process x(t) and E() the mean. The kurtosis k[x(t)] is:

$$K[x(t)]=\frac{Cum_{4}(x)}{E{\left(x^{2}\right)}^{2}}=\frac{E(x^{4})-3E{\left(x^{2}\right)}^{2}+12E{\left(x\right)}^{2}E(x^{2})-4E(x)E(x^{3})-6E{\left(x\right)}^{4}}{E{\left(x^{2}\right)}^{2}}$$

Assume the mean E() is zero, and k[p(x)] can be written as:

$$K[x(t)]=\frac{Cum_{4}(x)}{E{\left(x^{2}\right)}^{2}}=\frac{E(x^{4})}{E{\left(x^{2}\right)}^{2}}-3$$

Clearly, the kurtosis sign ks(x) is equal to the fourth-order cumulant sign. Some properties can be easily derived.

1) *Cum*_4_ (*ax*+ *b*) = *a*^4^*Cum*_4_ (*x*), so ks(x) is invariant by any linear transformation ks(ax+b)=ks(x)
2) Let *p*(*x*) = *p_e_* (*x*) + *p_o_* (*x*), where *p_e_* (*x*) is even and *p_o_* (*x*) is odd. It is easy to prove that ks(x) only depends on *p_e_* (*x*) and that *p_e_* (*x*) can be considered as a pdf.

Therefore, in the following, the study may be restricted to a zero-mean process x(t) whose the pdf p(x) is even and has a variance *σ*^2^*_x_* = 1

It is well known that the kurtosis of a Gaussian distribution is equal to zero. Intuitively, the sign of the kurtosis seems related to the comparison between p(x) and Gaussian distribution, by considering the asymptotic properties of the distribution and the following definition:

A pdf p(x) is said over-Gaussian (respectively sub-Gaussian), if ∀*x*≥*x*_0_, *p*(*x*) > *g*(*x*) (respectively, p(x)<g(x)), where g(x) is the normalized Gaussian pdf. In many examples, it seems that ks(x) is positive for over-Gaussian signals and negative for sub-Gaussian signals.

Let us consider that for x>0, the equation p(x)=g(x) only has one sulotion *p* > 0, it is known that the fourth-order cumulant of a Gaussian distribution is zero. As a consequence, we can write:

$\int_{-\infty }^{+\infty }x^{4}g(x)dx=3\int_{-\infty }^{+\infty }x^{2}g(x)dx=3$. In addition, we just may study the sign of $\gamma =\frac{1}{2}K[x(t)]$, and we can prove that

$$\gamma =\int_{0}^{\rho }x^{4}(p(x)-g(x))dx+\int_{\rho }^{\infty }x^{4}(p(x)-g(x))dx$$

Let us consider that the pdf p(x) is an over-Gaussian signal. Then, the sign of p(x)-g(x) remains constant on each interval [0,*ρ*] and [*ρ*, ∞]. Using the second mean value theorem, *γ* can be rewritten as:

$$\gamma =\lambda^{4}\int_{\rho }^{\infty }(p(x)-g(x))dx-\xi^{4}\int_{0}^{\rho }(g(x)-p(x))dx$$

Where 0 < *ξ* < *ρ* < *λ*. Using the fact that p(x) and g(x) are both pdf, we can deduce that

$$\int_{0}^{\infty }(p(x)-g(x))dx=\int_{0}^{\rho }(p(x)-g(x))dx+\int_{\rho }^{\infty }(p(x)-g(x))dx=0.$$

Taking into account that p(x) is over-Gaussian, we deduce

$$\int_{\rho }^{\infty }(p(x)-g(x))dx=\int_{0}^{\rho }(g(x)-p(x))dx>0$$

Using the above two equation, we remark that:

$$\gamma =(\lambda^{4}-\xi^{4})\int_{\rho }^{\infty }(p(x)-g(x))dx>0$$

Finally, if p(x) is an over-Gaussian pdf, then its kurtosis is positive. Using the same reason and under the same condition, we can claim that a sub-Gaussian pdf has a negative kurtosis.

There are some basic results about kurtosis given by Richard [12-14]. These results are helpful for understanding the statistical meaning of kurtosis. Here are some of these results.

For standard scores, $Z=\frac{X-\mu }{\sigma }$, the kurtosis of X is:

$$k=\frac{1}{N}\frac{\sum {\left(X-\mu \right)}^{4}}{s^{4}}=\frac{1}{N}{\sum \left(\frac{X-\mu }{s}\right)}^{4}=\frac{1}{N}\sum Z^{4}$$

Assume the two points of the distribution are at 0 and 1, with p being the frequency at 1. Then $\mu =p,\sigma =\sqrt{pq}$

$$\begin{array}{c} z_{1}=\frac{1-\mu }{\sigma }=\frac{q}{\sqrt{pq}}=\sqrt{\frac{p}{q}}\\ z_{0}=\frac{0-\mu }{\sigma }=\frac{-p}{\sqrt{pq}}=-\sqrt{\frac{p}{q}}\\ k=\frac{1}{N}\sum Z^{4}=(pz_{z}^{4}+qz_{0}^{4})=\frac{q^{2}}{p}+\frac{p^{2}}{q} \end{array}$$

As *p* + *q* =1

So we have

$$k=\frac{1}{N}\sum Z^{4}=(pz_{z}^{4}+qz_{0}^{4})=\frac{q^{2}}{p}+\frac{p^{2}}{q}=\frac{1}{pq}-3$$

For a three-point distribution in which the density is p, then

$$k=\frac{(\frac{1-p}{2}){\left(-1\right)}^{4}+p{\left(0\right)}^{4}+(\frac{1-p}{2}){\left(1\right)}^{4}}{\sigma^{4}}=\frac{\left(1-p\right)}{\sigma^{4}}$$

$$\sigma^{2}=(\frac{1-p}{2}){\left(-1\right)}^{2}+p{\left(0\right)}^{2}+(\frac{1-p}{2}){\left(1\right)}^{2}=1-p$$

So

$$k=\frac{1}{1-p}$$

Starting again with a normal distribution, moving scores from the tails and the center to the shoulders will decrease kurtosis. A uniform distribution certainly has a flat top, with *γ*_2_ = –1.2, but *γ*_2_ can reach a minimum value of –2 when two score values are equally probably and all other score values have probability zero (a rectangular U distribution, that is, a binomial distribution with n =1, p = 0.5). One might object that the rectangular U distribution has all of its scores in the tails, but closer inspection will reveal that it has no tails, and that all of its scores are in its shoulders, exactly one standard deviation from its mean.

Kurtosis is usually of interest only when dealing with approximately symmetric distributions. Skewed distributions are always leptokurtic [15]. Among the several alternative measures of kurtosis that have been proposed (none of which has often been employed), is one which adjusts the measurement of kurtosis to remove the effect of skewness [16].

There is much confusion about how kurtosis is related to the shape of distributions. Many people have asserted that kurtosis is a measure of the peakedness of distributions, which is not strictly true.

It is easy to confuse low kurtosis with high variance, but distributions with identical kurtosis can differ in variance, and distributions with identical variances can also differ in kurtosis. Here are some simple distributions that may explain what kurtosis is, in part, a measure of tail heaviness relatives to the total variance in the distribution.

A has the least kurtosis (–2 is the smallest possible value of kurtosis) and G the most. In the maximally platykurtic distribution A, which initially appears to have all its scores in its tails, no score is more than one σ away from the mean, that is, it has no tails! In the leptokurtic distribution G, which seems only to have a few scores in its tails, one must remember that those scores (5 and 15) are much farther away from the mean (3.3 σ) than are the 5's & 15's in distribution A. In fact, in G nine percent of the scores are more than three σ from the mean, much more than you would expect in a mesokurtic distribution (like a normal distribution), thus G does indeed have fat tails.

Kurtosis is the degree of peakedness of a distribution, defined as a normalized form of the fourth central moment of a distribution. The kurtosis for a number of some common distributions is shown below.

The following example makes it quite clear that a higher kurtosis implies that there are more extreme observations (or that the extreme observations are more extreme). It is also evident that a higher kurtosis also implies that the distribution is more ‘single-peaked’ (this would be even more evident if the sum of the frequencies was constant).

We may define mesokurtic as “having *β*_2_ equal to 3”, while platykurtic curves have *β*_2_ < 3, and leptokurtic *β*_2_ > 3. The important property which follows from this is that platykurtic curves have shorter “tails” than the normal curve of error and leptokurtic longer “tails”.

From the discussion above, the statistical meanings of kurtosis is given: kurtosis is a kind of measure of data's degree of outlier or data's peakedness.

## 3. The new application of kurtosis

The kurtosis of a random variable X is defined:

$$K=\frac{E(X-E(X))}{E(X-E(X))}$$

*x*_1_,…, *x_n_* is the samples from random variable X, and the kurtosis of samples is defined:

$$k_{n}=\frac{s_{n}^{4}}{{\left(s_{n}^{2}\right)}^{2}}=\frac{n\overset{n}{\underset{i=1}{\text{∑}}}{\left(x_{i}-\bar{x}\right)}^{4}}{{\left[\overset{n}{\underset{i=1}{\text{∑}}}{\left(x_{i}-\bar{x}\right)}^{2}\right]}^{2}}$$

Where $\bar{x}=\frac{1}{n}\overset{n}{\underset{i=1}{\text{∑}}}x_{i}$, $x_{n}^{4}=\frac{1}{n}\overset{n}{\underset{i=1}{\text{∑}}}{\left(x_{i}-\bar{x}\right)}^{4}$, $x_{n}^{2}=\frac{1}{n}\overset{n}{\underset{i=1}{\text{∑}}}{\left(x_{i}-\bar{x}\right)}^{2}$. It can be seen that the kurtosis of random variable and samples is independent of mean and variance.

The seismic signals of persons, trucks and tracklayers are collected at the sample rate of 1Ksps with the resolution of 16 bits, and the kurtosis extracted from each target signal is calculated every 512 samples. For each 512 samples of the signal, the kurtosis is calculated by the following formulation:

$$K_{x}=\frac{E\{{\left(x-\mu \right)}^{4}\}}{E^{2}\{{\left(x-\mu \right)}^{2}\}}$$

Where E denotes the mean of input signal, *μ* is referred to the mean of x.

### 3.1. Simulation results

Why are tailedness and peakedness both components of kurtosis? It is basically because kurtosis represents a movement of mass that does not affect the variance. Consider the case of positive kurtosis, where heavier tails are often accompanied by a higher peak. Note that if mass is simply moved from the shoulders of a distribution to its tails, then the variance will also be larger. To leave the variance unchanged, one must also move mass from the shoulders to the center, which gives a compensating decrease in the variance and a peak. For negative kurtosis, the variance will be unchanged if mass is moved from the tails and center of the distribution to its shoulders, thus resulting in light tails and flatness [17].

**Figure 1.**
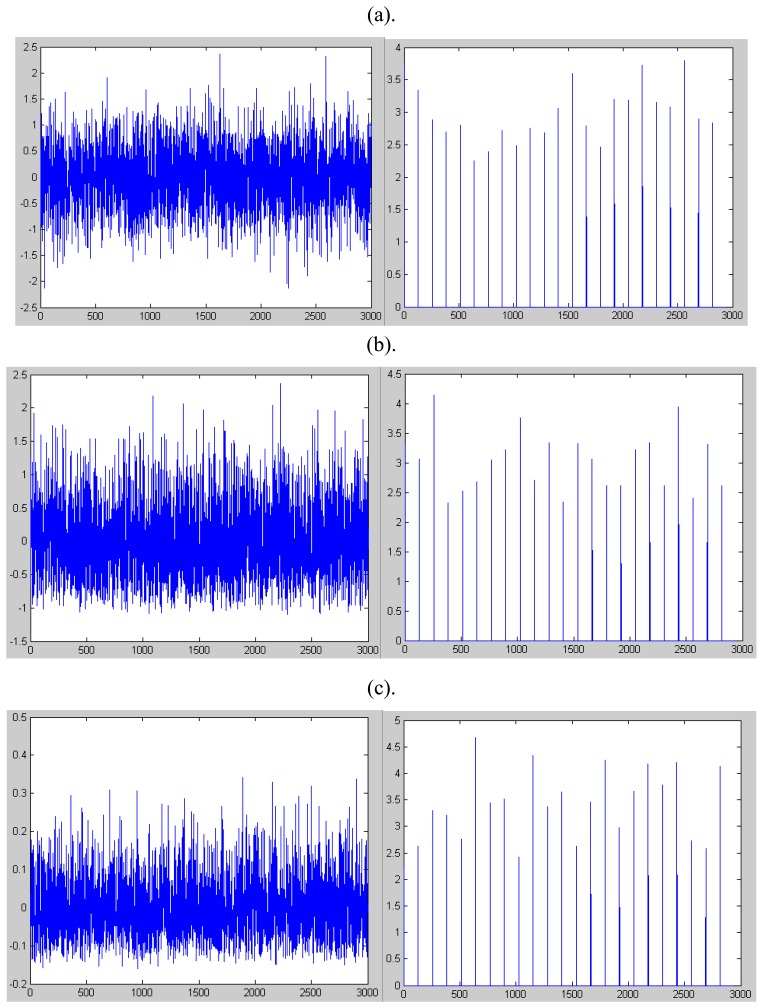
the kurtosis of several distributions, including normal distribution, rayleigh distribution and beta distribution. The left figure is the samples of the distribution, and the right is the kurtosis respectively. (a). the samples of normal distribution and its kurtosis. (b). the samples of rayleigh distribution and its kurtosis. (c). the samples of beta distribution and its kurtosis.

The kurtosis of several typical distributions, including normal distribution, rayleigh distribution and beta distribution, is given in figure 1.

### 3.2. Kurtosis for background noise, tracklayer and truck

In this section, we will simulate the results of kurtosis. First, we collect the seismic signal by the seismic sensors. The raw seismic signal is then divided into N blocks with 512 samples each. The parameter of kurtosis is calculated every block. That is to say, we can get only one value from 512 samples. In order to make the simulation results clearer and easier to understand, we add 511 zeros to each kurtosis to form the final simulation results.

**Figure 2.**
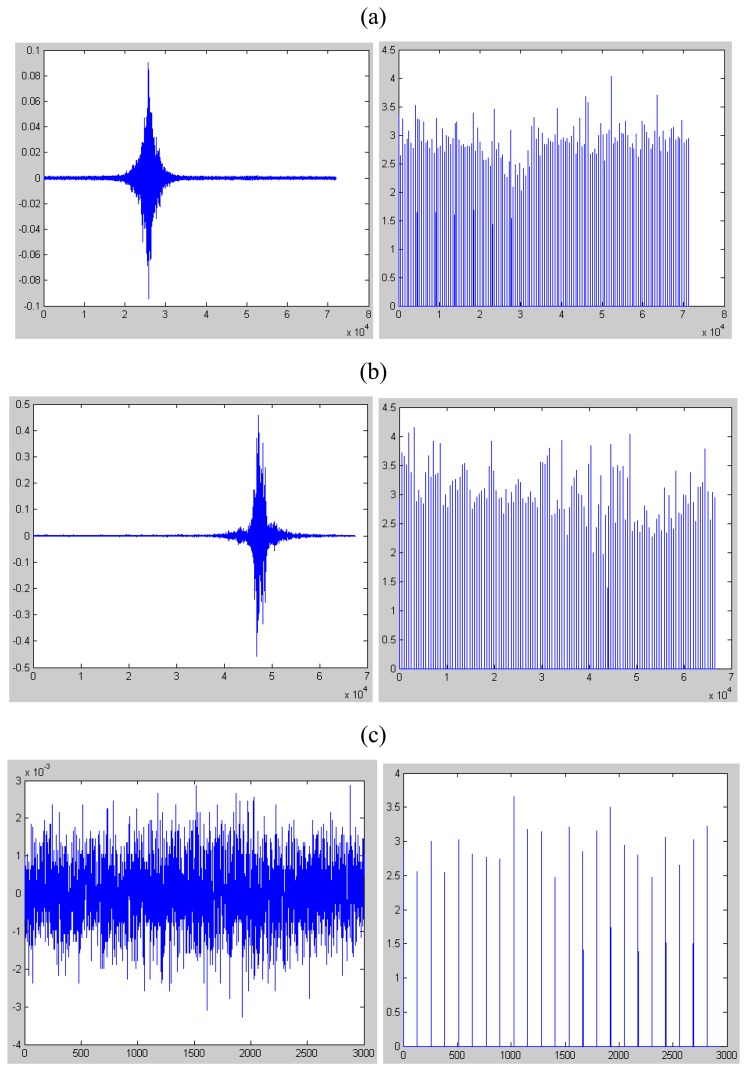
The seismic signal, collected in gravelly clay region, of target(left) and its kurtosis every 512 samples. The left figure of (a) is the seismic signal of tracklayer and the right figure of (a) is the kurtosis calculated every 512 samples. The left figure of (b) is the seismic signal of truck and the right figure of (b) is the kurtosis calculated every 512 samples too. The left of (c) is the seismic signal of truck and the right is the kurtosis calculated every 512 samples too.

**Figure 3.**
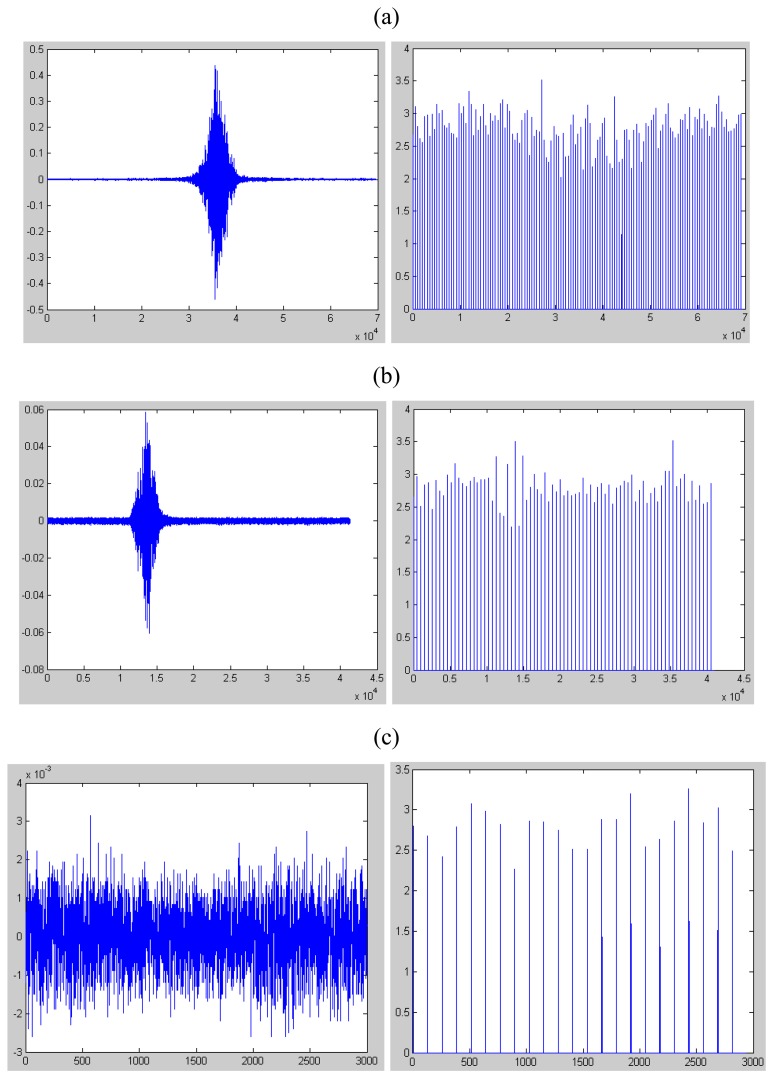
The seismic signal, collected in loessal soil region, of target(left) and its kurtosis every 512 samples. The left figure of (a) is the seismic signal of tracklayer and the right figure of (a) is the kurtosis calculated every 512 samples. The left figure of (b) is the seismic signal of truck and the right figure of (b) is the kurtosis calculated every 512 samples too. The left of (c) is the seismic signal of truck and the right is the kurtosis calculated every 512 samples too.

In figure 2 and figure 3, we list the seismic signal of tracklayer, light truck and background noise deprived in gravelly clay region and loessal soil region respectively. Also, we plot the parameter of kurtosis of each target.

From figure 2, we can see that the parameter of kurtosis of background noise environment is far below 5 while the value of tracklayer and truck signal rises but is still below 5 in gravelly clay region.

From figure 3, we can see that the parameter of kurtosis of background noise environment is also far below 5 while the value of tracklayer and truck signal rises but is still below 5 in loessal soil region.

After comparing the results from figure 2 and figure 3, we can see that the kurtosis of the non-impulsive signal is below 5 no matter in which type of geologic features. In another words, the algorithm we use needs no machine study or training which is quite useful and convenient when we apply it in any new atmosphere.

### 3.3. Kurtosis for person

**Figure 4.**
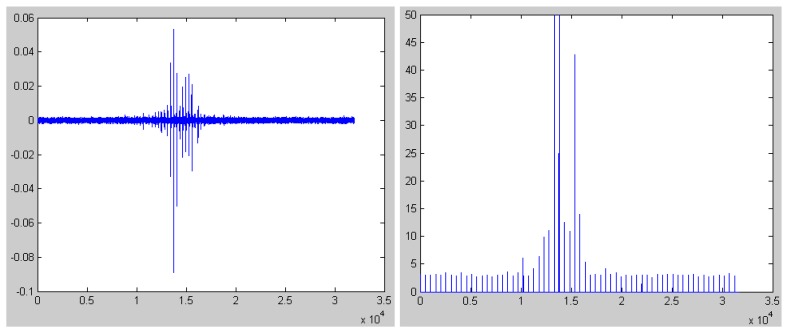
The simulation result of the seismic signal of person in gravelly clay region.

**Figure 5.**
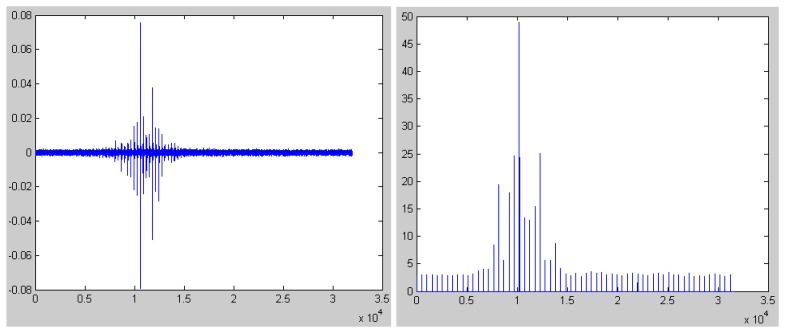
The simulation result of the seismic signal of person in loessal soil region.

In figure 4 and figure 5, we give the seismic signal of person deprived in gravelly clay region and loessal soil region respectively. Also, we plot the parameter of kurtosis.

It can be seen from figure 4 and figure 5 that the parameter of kurtosis is below 5 when there is no person and the results are in accordance with the results in figure 2 and figure 3. Oppositely, the value of kurtosis is far beyond 4 when some person passes by. Also, we can see the adaptation of the algorithm in different region from the comparison between figure 3 and figure 4.

From above, we can make the following conclusions:

1) The kurtosis of impulsive signals is far beyond 5;
2) The kurtosis of non-impulsive signals is below 5;
3) The values of kurtosis are independent of the geologic features and are only dependent on the feature of signals.

From the analysis above, it is clear that we can distinguish person from other targets depending on the value of kurtosis in any atmosphere and needs no machine study and training.

## 4. Conclusion

From the discussion above, it is clear that walker can be detected and distinguished from other targets by comparing the kurtosis of the seismic signal. The value of kurtosis depends on the features of the signals and is independent of the geologic features.

## Figures and Tables

**Table 1.** Kurtosis for 7 Simple Distributions Also Differing in Variance.

**X**	**freq A**	**freq B**	**freq C**	**freq D**	**freq E**	**freq F**	**freq G**
05	20	20	20	10	05	03	01
10	00	10	20	20	20	20	20
15	20	20	20	10	05	03	01

Kurtosis	−2.0	−1.75	−1.5	−1.0	0.0	1.33	8.0

Variance	25	20	16.6	12.5	8.3	5.77	2.27

**Table 2.** The kurtosis for a number of common distributions.

**Distribution**	**Kurtosis excess**
Bernoulli distribution	$\frac{1}{1-p}+\frac{1}{p}-6$
Beta distribution	$\frac{6[a^{3}+a^{2}(1-2b)+b^{2}(1+b)-2ab(2+b)]}{ab(2+a+b)(3+a+b)}$
Binomial distribution	$\frac{6p^{2}-6p+1}{np(1-p)}$
Chi-squared distribution	$\frac{12}{r}$
Fisher-Tippett distribution	$\frac{12}{5}$
Gamma distribution	$\frac{12}{a}$
Geometric distribution	$5-p+\frac{1}{1-p}$
Half-normal distribution	$\frac{8(\pi -3)}{{\left(\pi -2\right)}^{2}}$
Laplace distribution	3
Log normal distribution	*e*^4*S*^2^^ + 2*e*^3*S*^2^^ + 3*e*^2*S*^2^^ −6
Maxwell distribution	$-\frac{4(96-40\pi +3\pi^{2})}{{\left(3\pi^{2}-8\right)}^{2}}$
Negative binomial distribution	$\frac{6-p(6-p)}{r(1-p)}$
Normal distribution	0
Poisson distribution	$\frac{1}{v}$
Rayleigh distribution	$\frac{6\pi (4-\pi )-16}{{\left(\pi -4\right)}^{2}}$
Student's t-distribution	$\frac{6}{n-4}$
Continuous uniform distribution	$-\frac{6}{5}$
Discrete uniform distribution	$-\frac{6(n^{2}+1)}{5(n^{2}-1)}$

**Table 3.** Kurtosis for Seven Simple Distributions Not Differing in Variance.

**X**	**Freq. A**	**Freq. B**	**Freq. C**	**Freq. D**	**Freq. E**	**Freq. F**	**Freq. G**
–6.6	0	0	0	0	0	0	1
–0.4	0	0	0	0	0	3	0
1.3	0	0	0	0	5	0	0
2.9	0	0	0	10	0	0	0
3.9	0	0	20	0	0	0	0
4.4	0	20	0	0	0	0	0
5	20	0	0	0	0	0	0
10	0	10	20	20	20	20	20
15	20	0	0	0	0	0	0
15.6	0	20	0	0	0	0	0
16.1	0	0	20	0	0	0	0
17.1	0	0	0	10	0	0	0
18.7	0	0	0	0	5	0	0
20.4	0	0	0	0	0	3	0
26.6	0	0	0	0	0	0	1

Kurtosis	−2.0	−1.75	−1.5	−1.0	0.0	1.33	8.0

Variance	25	25.1	24.8	25.2	25.2	25.0	25.1
